# Supplementary material for: Combined Photothermal Therapy and Lycium barbarum Polysaccharide for Topical Administration to Improve the Efficacy of Doxorubicin in the Treatment of Breast Cancer
Source: Pharmaceutics. 2022 Dec 1;14(12):2677. doi: 10.3390/pharmaceutics14122677 (PMC9785128; doi:10.3390/pharmaceutics14122677)
Supplement: Supplementary file 1 [file pharmaceutics-14-02677-s001.zip › pharmaceutics-2042265-supplementary.pdf]

Supplementary Materials

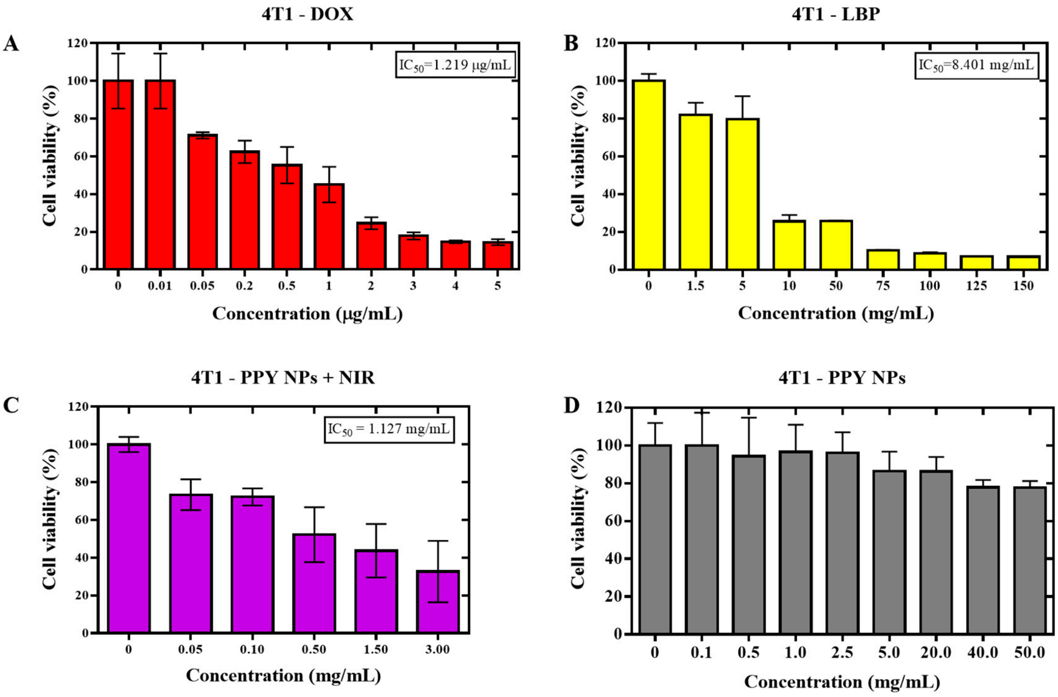

Figure S1: Cell viability of DOX, LBP, PPY NPs + NIR, PPY NPs against 4T1 cell line for incubation 72 h at 37°C, n = 5.

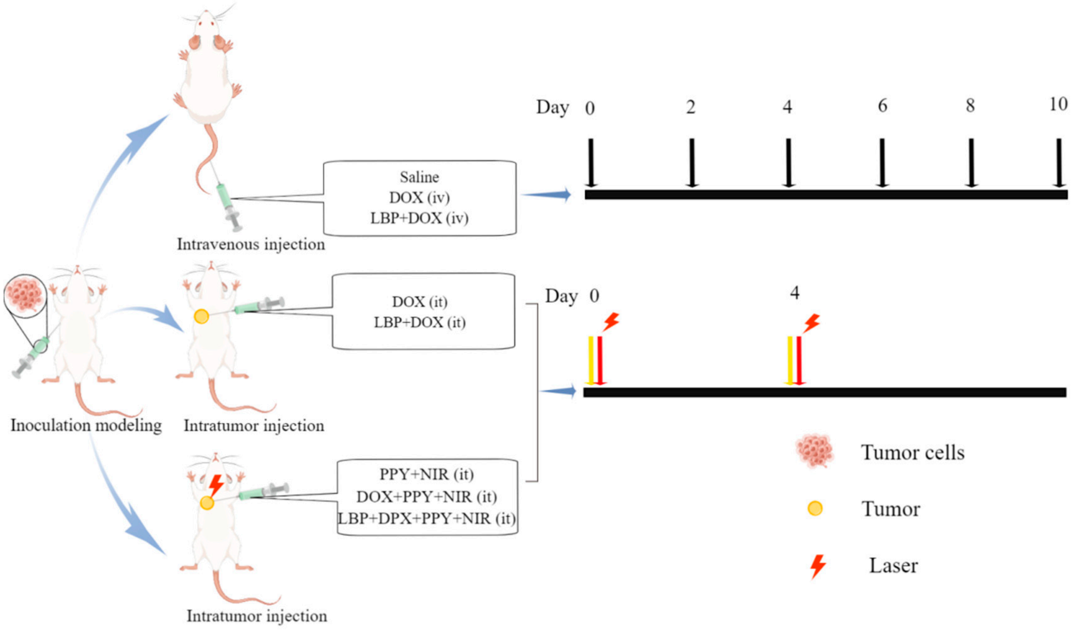

Figure S2: Treatment schedule scheme: intravenous injection and intratumoral injection (This picture was drawn by Figdraw.);

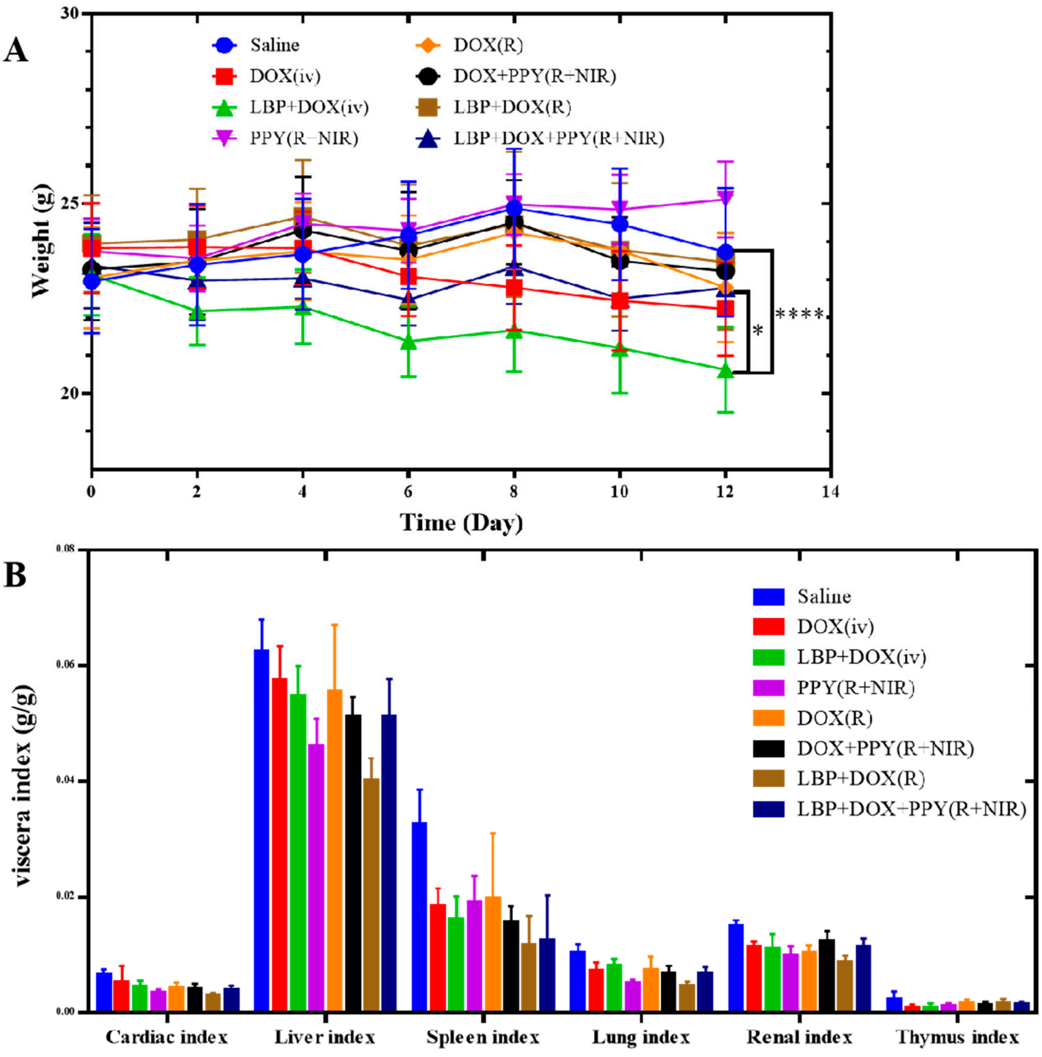

Figure S3: Changes of body weight (A) (mean ± SD, n = 6); Organ index of 8 groups of animals, n = 6. (B).
